# Predictors for wound healing complications and prolonged hospital stay in patients with isolated calcaneal fractures

**DOI:** 10.1007/s00068-021-01863-1

**Published:** 2022-01-06

**Authors:** Felix Marius Bläsius, Laura Elisabeth Stockem, Matthias Knobe, Hagen Andruszkow, Frank Hildebrand, Philipp Lichte

**Affiliations:** 1 Department of Orthopaedics, Trauma and Reconstructive Surgery, University Hospital RWTH Aachen, Pauwelsstraße 30, 52074 Aachen, Germany; 2 Department of Orthopaedic and Trauma Surgery, Lucerne Cantonal Hospital, Spitalstrasse 16, 6000 Lucerne, Switzerland

**Keywords:** Calcaneal fracture, Wound healing, Length of stay, Open fracture

## Abstract

**Purpose:**

Surgically treated calcaneal fractures have a high risk of postoperative wound healing complications and a prolonged length of hospital stay (LOS). The aim of this study was to identify predictor variables of impaired wound healing (IWH) and LOS in surgically treated patients with isolated calcaneal fractures.

**Methods:**

This retrospective cohort study analyzed data on patients aged 18 years or older who were admitted to a level I trauma center with isolated calcaneal fractures between 2008 and 2018. Multivariable regression models were used to identify predictor variables.

**Results:**

In total, 89 patients (age: 45.4 years; SD: 15.1) were included. In 68 of these patients, low-profile locking plate osteosynthesis was performed, and a minimally invasive approach (MIA) (percutaneous single screws/K-wire or low-profile locking plating via a sinus tarsi approach) was applied in 21 patients. Multivariable regression analysis revealed that a higher preoperative Böhler’s angle (*β* = − 0.16 days/degree, 95% CI [− 0.25, − 0.08], *p* = 0.004) and MIA (*β* = − 5.04 days, 95% CI [− 8.52, − 1.56], *p* = 0.002) reduced the LOS. A longer time-to-surgery (*β* = 1.04 days/days, 95% CI [0.66, 1.42] *p* = 0.001) and IWH increased the LOS (*β* = 7.80 days, 95% CI [4.48, 11.12], *p* = 0.008). In a subsequent multivariable regression analysis, two variables, open fractures (OR: 14.6, 95% CI [1.19, 180.2], *p* = 0.030) and overweight (BMI > 24) (OR: 3.65, 95% CI [1.11, 12.00], *p* = 0.019), increased the risk of IWH.

**Conclusion:**

Advanced treatment algorithms for open fractures are needed to reduce the risk of IWH.

**Supplementary Information:**

The online version contains supplementary material available at 10.1007/s00068-021-01863-1.

## Introduction

Fractures of the calcaneus are of major socioeconomic importance, as individuals of working age (30–60 years) are the highest at-risk group [1]. Intra-articular calcaneal fractures present a particular challenge, as they are associated with a poor outcome. Calcaneal fractures are caused by a high-energy mechanism, which often results in complex fracture patterns, with a high risk of post-traumatic arthrosis and chronic pain, with a need for subsequent arthrodesis. With the aim of achieving the best possible reconstruction of the 3-dimensional anatomy, internal fixation using low-profile locking plates has become the preferred treatment approach for calcaneal fractures in recent decades [2]. There is evidence for improved functional outcomes using this approach, which is associated with potential economic benefits due to reduced rehabilitation times and lower rates of secondary osteoarthrosis and arthrodesis [1].

After osteosynthesis of closed calcaneal fractures, wound healing complications are common, affecting up to 25% of patients [3, 4]. The risk of wound healing complications increases (up to 30%) in cases of surgical treatment of open fractures [5]. Independent of the fracture type, the type of osteosynthesis influences the incidence of wound healing complications, with a higher absolute risk in cases of open reduction and internal fixation (ORIF) compared to a minimally invasive approach (MIA) [6, 7]. Postoperative complications can prolong the length of hospital stay (LOS) and extend rehabilitation periods and consequently lead to higher costs for the health care system. To ensure the fastest possible recovery of foot function, to manage patient expectations, and to identify patients at risk of postoperative wound healing disorders and subsequent prolongation of LOS, the variables influencing recovery of foot function need to be identified.

The aim of this study was to identify predictor variables of impaired wound healing (IWH) and prolongation of LOS in surgically treated patients with isolated calcaneal fractures.

## Patients and methods

### Ethics

This retrospective case–control study was approved by the institutional review board of RWTH Aachen University (independent ethics committee at the RWTH Aachen Faculty of Medicine, registration no. EK146/19). The study protocol was registered a priori at the Center for Translational & Clinical Research of the RWTH Aachen Faculty of Medicine (registration no. 19-048).

### Selection of patients

**Fig. 1:**
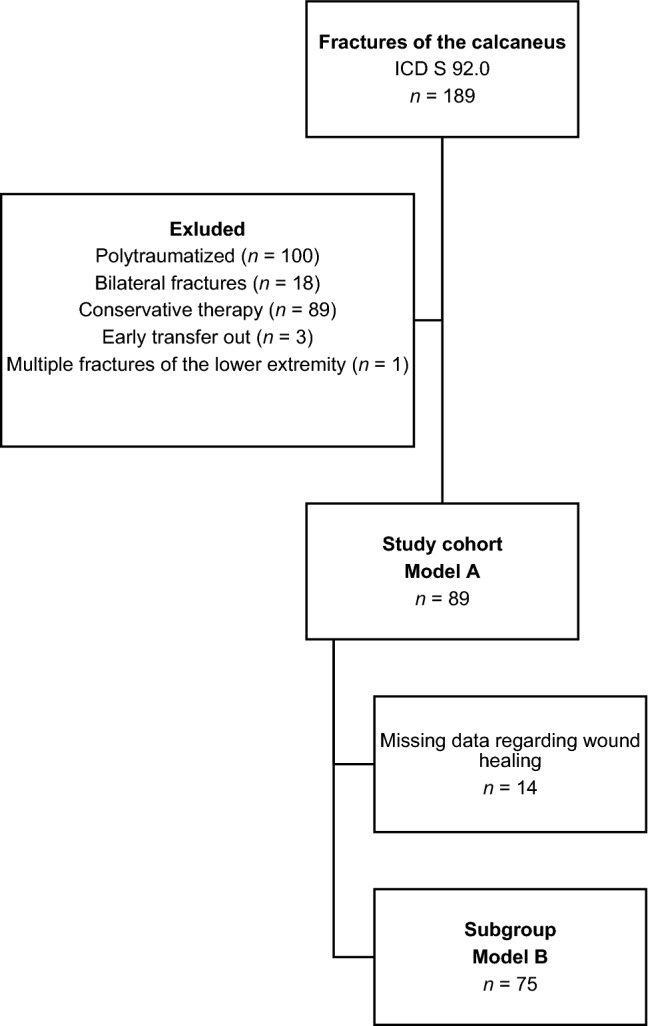
Flow diagram illustrating the selection of patients

Data on adults with isolated calcaneal fractures were retrospectively obtained from the hospital information system of a German academic level I trauma center in Aachen (also known as Aix-la-Chapelle), Germany. The inclusion criteria were (Fig. 1)as follows:

- Surgically treated isolated fracture of the calcaneus
- Age ≥ 18 years
- Availability of data regarding the treatment modality
- Treatment between 2008 and 2018

The exclusion criteria were as follows:

- Early hospital release

### Coding of the variables

Information on the coding of the variables can be found in Supplement 1.

### Definitions

Fixation by implantation of low-profile locking plate osteosynthesis using an extensile lateral approach was defined as ORIF. Closed reduction and minimally invasive internal fixation using single screws/K-wires or minimally invasive plating via a sinus tarsi approach was defined as MIA. Overweight was defined as a body mass index (BMI) of > 24. Böhler’s angle was determined as described previously [8]. Fracture morphology was classified according to Sanders’ classification [9]. Smokers were defined as patients who reported actively smoking on admission. IWH diagnosis criteria included non timely wound healing 14 days postoperatively and at least one of the following: (A) purulent drainage; (B) signs or symptoms of an infection: progredient pain or tenderness, swelling, redness, heat; (C) laboratory confirmation of a SIRS in the absence of another focus; (D) wound dehiscence.

### Treatment algorithm

The decision of whether surgery was done through an ORIF or MIA was made by clinical criteria. Criteria for the use of a MIA were multifactorial and the decision to use or not to use MIA was made by a specialist. Criteria included soft tissue swelling, obesity, pre-existing conditions, compliance, and preexisting conditions (e.g., active smoker, drug abuse, diabetes mellitus, etc.). The timing of ORIF was determined based on periodic soft tissue assessments (e.g., wrinkle sign test) by a specialist. Follow-up treatment included unloading of the injured extremity for 10 weeks in a fracture orthosis boot. From the start of the 11th week, patients began step-by-step loading in a fracture orthosis boot (Settner’s orthosis) in the following 4 weeks until full load. The diagnosis of IWH was made by a medical staff member with specialist standard at the Department of Orthopaedics, Trauma and Reconstructive Surgery.

### Statistics

Continuous values are presented as mean, standard deviation (SD), and median, where applicable. Differences in categorical and continuous variables were evaluated by a *χ*^2^ test and Wilcoxon’s rank-sum test, respectively. The significance level was set at *α* = 0.05 (two-sided *p* value). All statistical analyses were performed using the Statistical Package for Social Sciences (SPSS 27.0; IBM Inc., Armonk, NY, USA).

### Model A: multivariable linear regression model

We fitted a full multivariable logistic regression model, using LOS as the dependent endpoint. To identify independent variables for inclusion in the multivariable linear regression model, univariable linear regression analyses of multiple variables were conducted (Supplement 2). The variables explored included age, sex, time-to-surgery (TTS), open fracture, MIA, IWH, Böhler’s angle, ASA physical status classification system, and overweight. Coding and the cases per variable are presented in Supplement 1. The accepted level of statistical significance in the multivariable regression was set at *α* = 0.05. The significance of each regression coefficient was analyzed by a *t*-test. The significance level was set at *α* = 0.05. The results are reported as odds ratios (ORs) with their 95% confidence intervals (CIs). CIs were generated using the bootstrap method (1000 replications). *R*^2^ was calculated as a measure of the goodness of fit of the models.

### Model B: multivariable binary logistic regression model

The binary logistic regression model was fit using the dichotomous variable “IWH” as the dependent endpoint. As with model A, univariable binary logistic regression analyses of multiple variables were conducted to identify independent variables for inclusion in the logistic regression model (Supplement 3). The variables included age, sex, TTS, open fracture, MIA, active smoker, alcohol abuse, drug addiction, arterial hypertonia, anticoagulation, and overweight (Supplement 2). The significance level was set at *α* = 0.20. The statistical significance of each regression coefficient was tested by the Wald test. The significance level was set at *α* = 0.05. CIs were generated using the bootstrap method (1000 replications). The goodness of fit was measured using ROC_AUC_ and the Hosmer–Lemeshow test (*X*^2^).

### Validation of the models

We studied one additional data set for validation: all patients with an isolated calcaneal fracture treated in the same academic level I trauma center from 2019 to 2021 (Supplement 4). This cohort was selected due to a change in personnel of the senior surgeon, so a corresponding bias could be excluded. Validation of the multivariable binary logistic regression model was performed by computing the ROC_AUC_ of the model in the validation cohort. Validation of the linear regression was performed by analyzing Pearson’s correlation between the results of model B and the patient’s LOS.

## Results

The demographics of the 89 patients included in the study cohort are displayed in Table 1. The mean age was 45.4 (SD: 15.1) years.

**Table 1:** Characteristics of patients in the study cohort

Independent variables	Study cohort *n* = 89
Age, mean, median (SD)	45.4, 47.0 (15.1)
Male, *n*	76
Sanders classification, *n*	
Type I	0
Type II	4
Type III	66
Type IV	19
Open fracture	5
TTS^1^, mean, median (SD)	3.9, 3.0 (3.9)
LOS^2^, mean, median (SD)	16.0, 13.0 (9.2)
ORIF^3^, *n*	68
BMI > 24, *n*	21
Drug abuse, *n*	10
Diabetes, *n*	2
Active smoker	30
Alcohol abuse, *n*	13
Impaired wound healing	21

^1^Time-to-surgery

^2^Length of stay

^3^Open reduction and internal fixation

### Model A: hospital LOS

The univariable analyses identified the preoperative Böhler’s angle, TTS, surgical treatment method, and IWH as independent variables in Model A (Supplement 2).

In the multivariable regression analysis, a higher preoperative Böhler’s angle (*β* = − 0.163 days/degree, 95% CI [− 0.247, − 0.079], *p* = 0.004), and MIA (*β* = − 5.040 days, 95% CI [− 8.522, − 1.557], *p* = 0.002) were independent variables reducing the LOS. A longer TTS (*β* = 1.040 days/days, 95% CI [0.663, 1.417] *p* = 0.001) and IWH were independent variables increasing the LOS (*β* = 7.800 days, 95% CI [4.482, 11.117], *p* = 0.008) (Table 2). In our cohort, the predictive validity of the model for LOS was moderate (*R*^2^ = 0.562).

**Table 2:** Multivariable linear regression model with the hospital length of stay as the dependent variable

Independent variables	Regression coefficient *β*	*p* value	95% CI	Collinearity	
				**Tolerance**	**VIF**
Constant	17.260		12.099, 22.422		
Böhler’s angle	− 0.163	0.004	− 0.247, − 0.079	0.885	1.074
TTS^1^	1.040	0.001	0.663, 1.417	0.931	1.096
MIA^2^	− 5.040	0.002	− 8.522, − 1.557	0.894	1.119
Impaired wound healing	7.800	0.008	4.482, 11.117	0.925	1.081

*R*^2^ was 0.562

^1^Time-to-surgery

^2^Minimally invasive approach

### Model B: IWH

The univariable analyses identified the variables open fractures, overweight, surgical treatment method, and alcohol abuse as independent variables in Model B (Supplement 3).

**Fig. 2:**
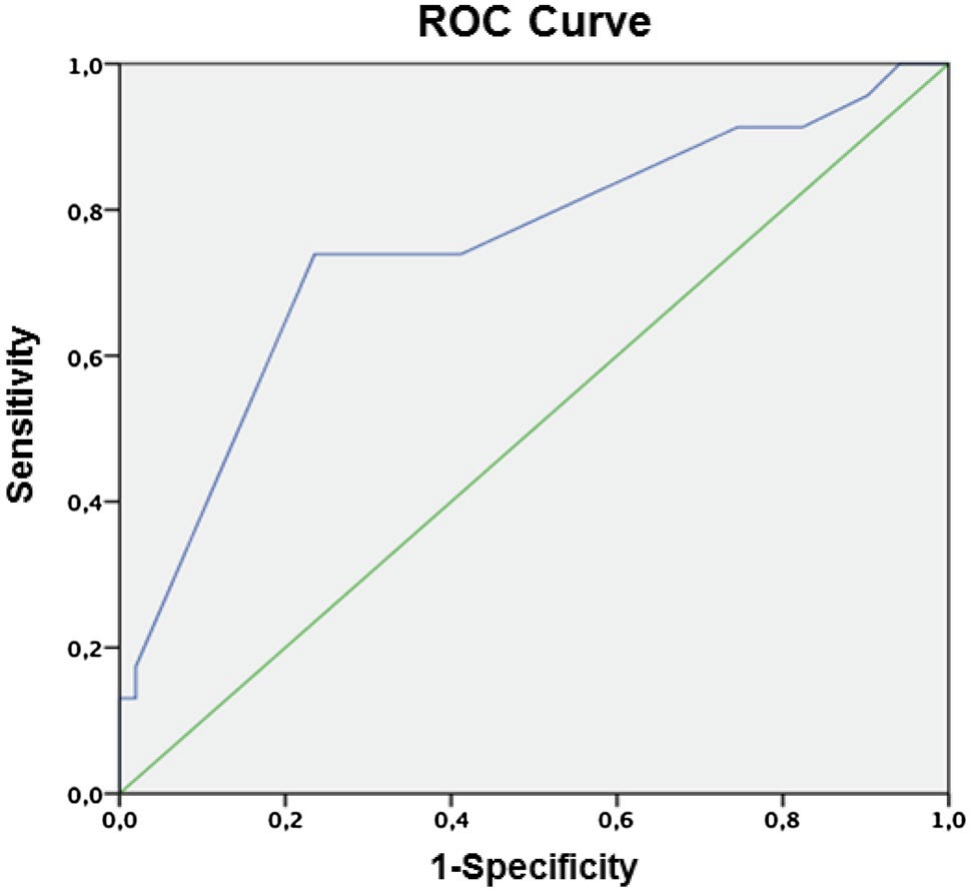
ROC_AUC_ curve of Model B

Open fractures (OR: 14.645, 95% CI [1.190, 180.15], *p* = 0.030) and overweight (BMI > 24) (OR: 3.647, 95% CI [1.109, 11.995], *p* = 0.019) were independent variables increasing the risk of IWH (Table 3). The predictive validity of the model for IWH was high (ROC_AUC_ = 0.749, 95% CI [0.620, 0.878]; Fig. 2).

**Table 3:** Multivariable binary logistic regression model with impaired wound healing as the dependent variable

Independent variables	Regression coefficient *β*	Wald	*p* value	OR	95% CI
Open fracture	2.684	4.394	0.030	14.645	1.190, 180.15
Alcohol abuse	− 1.477	1.733	0.090	0.228	0.025, 2.059
MIA^1^	− 1.114	2.354	0.094	0.328	0.079, 1.362
BMI > 24^2^	1.294	4.537	0.019	3.647	1.109, 11.995

ROC_AUC_ was 0.749, 95% CI [0.620, 0.878], Hosmer–Lemeshow-Test was not statistically significant (*X*^*2*^ value = 4.679, df = 4, *p* = 0.322)

^1^Minimally invasive approach

^2^BMI > 24

### Validation data set

The models were tested in a validation data set comprising 25 patients (characteristics: Supplement 4). The models showed consistent predictive validity (Model A: ROC_AUC_ = 0.69, 95% CI [0.449, 0.932]; Model B: *β* = 0.586, *p* = 0.002).

## Discussion

In this retrospective study, a higher preoperative Böhler’s angle and MIA shortened the LOS. In contrast, IWH led to an increase in the LOS. The TTS prolonged the LOS only by the corresponding amount of time. Two variables, overweight and open fractures, were the main risk factors for IWH.

In preoperative soft tissue monitoring of patients with isolated calcaneal fractures, the extent of soft tissue swelling is the most important criterion for operability [2]. The finding that the TTS extended the total stay only by the corresponding amount of time suggests that current soft tissue monitoring practice is effective and does not prolong the postoperative LOS. It can be assumed that waiting for the swelling to subside reduces the number of complications and avoids longer postoperative stays due to the absence of complications. However, due to a lack of randomized studies, this assumption is speculative.

Böhler’s angle, a surrogate for the amount of energy absorbed by the foot, is considered a reliable tool for estimating the severity of the injury in calcaneal fractures and for estimating patient satisfaction 2 years post-trauma [10–12]. Previous studies have demonstrated a relationship between high-energy injury mechanisms and fracture complexity or accompanying soft tissue damage in various types of fractures, including calcaneal fractures and fractures of the ankle [13, 14]. In such cases, prolonged pre- and postoperative soft tissue monitoring or further surgical interventions may be required. High-energy mechanisms and resulting complex fractures may explain the observed independent relationship of the LOS with Böhler’s angle in the present study.

The findings of the present study that ORIF using a low-profile locking plate resulted in a prolonged stay compared to MIA was expected. While MIA could be performed promptly after trauma, ORIF requires critical soft tissue evaluation and waiting for the swelling to resolve. Waiting for the resolution of soft tissue swelling is a well-accepted international standard in fracture patients [2].

The major impact of wound healing disorders on the LOS in the present study was also expected. We demonstrated this relationship after correction for various confounders. In our study, the LOS was prolonged in those with a wound healing disorder by almost 8 days. This finding emphasizes the enormous socioeconomic significance of this complication and the importance of wound healing in the patient's recovery. For these reasons, there is an urgent need to identify independent risk factors for IWH while considering possible confounders.

In our study, the risk of IWH increased 3.6 and 14.6 times in the presence of two independent risk factors “overweight” and “open fractures,” respectively. The association between open fractures and wound healing disorders has been investigated in detail in the literature [15]. In common with the present study, Wang et al. reported a comparably high OR of 9.48 for open fractures in a multivariable analysis, which included 681 patients with calcaneal fractures [16]. The results of their study showed a similar trend to that observed in our study and indicated that open fractures are the main risk factor for IWH. Unlike our study, patients with multiple fractures, including bilateral fractures, were included in the study by Wang et al. [16]. Thus, the results of the two studies are comparable only to a limited extent. Another reason for the 14-fold increase in the risk of wound healing disorders in the present study may be due to the baseline data. Our cohort included a high proportion of complex and open fractures. Moreover, the trauma center in the present study is a level I center and thus a referral center for complex injuries, as evidenced by the high proportion of Sanders III and IV fractures (95%) in our cohort. There is good evidence that complex injuries are associated with severe soft tissue damage and open fractures due to the underlying high-energy impact [10, 12].

The second major risk factor for wound healing disorders in our study was overweight. Numerous retrospective case–control studies have described BMI as a risk factor for wound infection after a calcaneus fracture [17, 18]. Overweight is a major challenge in surgery. Pierpont et al. [19] reported that it has a negative impact on wound healing and that it is associated with wound infections in numerous surgical procedures. In their study, the effect of obesity on local wound healing was primarily attributed to a decrease in elastin, an increase in collagen V and VI, and lower capillary density, with accompanying relative tissue hypoxia, impaired angiogenesis due to chronic low-grade inflammation, and micronutrient deficiencies [19]. In addition, Knobe et al. [20] demonstrated in a prospective study of 34 patients that preoperative oxygen saturation at the surgical site could be a predictor of wound revision after surgical treatment of a calcaneal fracture. Therefore, chronic inflammation and impaired angiogenesis may explain the increase in wound healing disorders in the presence of a high BMI.

Surprisingly, ORIF was not a risk factor for wound healing disorders in our study. There are few prospective studies on the association of ORIF with IWH. Jin et al. and Kumar et al. conducted randomized trials on small numbers of cases and demonstrated lower rates of IWH in MIA than ORIF groups [21, 22]. However, the focus of these studies was to investigate surgical site infections after surgical therapy of closed calcaneal fractures and only a few confounding factors have been taken into account. For example, the BMI or nicotine abuse were not recorded and statistical analyses were not adjusted for these factors. In addition, there is a lack of reporting of power analyses, which means that statistical results lack reliability. In addition, the high proportion of complex fractures with large soft tissue damage in our cohort may have neutralized the weaker effect of ORIF. In addition, Peng et al. [23] demonstrated in a meta-analysis that the commonly used sinus tarsi approach is associated with a significantly lower risk for wound infections compared with the extensile lateral approach. However, very heterogeneous studies since 1995 were included for this purpose, and their comparability must be critically questioned.

Various reasons could explain our observations that ORIF did not lead to increased IWH rates in our study compared with MIA. Surgical techniques and hygiene concepts have undergone significant developments since the 1990s and early 2000s and might have led to a reduction in incidences of IWH [6]. Modern implants, such as low-profile locking plates, have been shown to provide improved stability compared to older implants [24, 25]. As good balance between biomechanical stability and micromovements is a key element for successful secondary fracture and primary wound healing, modern implants may have contributed to a reduction in complication rates, including IWH [26]. Unfortunately, as no randomized studies comparing implants of different generations exist, the potential relationship between implant type and reduced complications rates remains unclear.

Our findings on the association of BMI with wound infections and IWH were in accordance with those of Su et al. [18]. However, two variables (active smoker and TTS) were confirmed as risk factors for wound infections and IWH in Su et al.’s study but not in our study. Some possible reasons for the discord in the findings can be conjectured. First, Su et al. excluded open fractures in their study and focused primarily on surgical site infections. Conceivably, the inclusion of open fractures in our study as an independent variable may have masked the weaker influence of nicotine abuse and TTS on wound healing. Second, in view of the width of our CIs and the small number of affected patients (diabetes: *n* = 2), our study ultimately lacks power regarding this risk factor and is subject to a reasonable degree of statistical uncertainty. Due to the small number of cases in the center, a reliable estimation of the influence of diabetes will only be possible by a meta-analysis or a multicenter study.

In summary, IWH was identified as a major risk factor for prolongation of LOS in our study. Two variables, open fractures and overweight, were risk factors for wound healing disorders. Improved management approaches for open fractures and validation of these approaches in randomized trials as well as a more profound understanding of the influence of a high BMI on wound healing are urgently needed.

## Strengths and limitations

In terms of strengths, against the background of a satisficing rather than an optimizing concept, our models can be considered to have excellent clinical applicability and sufficient statistical validity [27]. In addition, the results were validated using a validation data set from our institution. In terms of limitations, wide CIs due to the relatively small number of cases point to some statistical uncertainty. Our results need to be validated using a multicenter approach, with a large number of cases. Another limitation is the retrospective design of the study, so that only correlations, but no causal relationships, can be discussed. The individual entities grouped under IWH (e.g. wound dehiscence, delayed wound healing, surgical site infections, deep wound infections) could not be disaggregated because of the retrospective design.

## Conclusion

A prolonged hospital stay after a calcaneal fracture has significant consequences for patients in terms of hospital costs and time off work. The single most important factor responsible for an extended LOS is IWH. Overweight and open fractures predispose patients to wound healing disorders, regardless of the surgical procedure performed. Improved management approaches for open fractures and validation of these approaches in randomized trials, as well as a more profound understanding of the influence of overweight on wound healing, are urgently needed.

## Supplementary Information

Below is the link to the electronic supplementary material.

Supplementary file1 (DOCX 35 KB)

## Data Availability

The data are available from the corresponding author upon request.

## Abbreviations

BMI: Body mass index
CI: Confidence interval
LOS: Length of stay
MIA: Minimally invasive approach
OR: Odds ratio
ORIF: Open reduction and internal fixation
SD: Standard deviation
TTS: Time-to-surgery
