# Supplementary material for: Predictors for wound healing complications and prolonged hospital stay in patients with isolated calcaneal fractures
Source: Eur J Trauma Emerg Surg. 2022 Jan 6;48(4):3157–63. doi: 10.1007/s00068-021-01863-1 (PMC9360130; doi:10.1007/s00068-021-01863-1)
Supplement: Supplementary file 1 — Supplementary file1 (DOCX 35 KB) [file 68_2021_1863_MOESM1_ESM.docx]

## Supplement 1

Coding of the variables analyzed.

| **Independent variables** | **Coding** |
| --- | --- |
| **Age** | Metric, days |
| **Sex** | 1=male, 2=female |
| **TTS^1^** | Metric, days |
| **Open Fracture** | 0=yes, 1=no |
| **MIA^2^** | 2=MIA, 1=ORIF |
| **Impaired wound healing** | 0=no, 1=yes |
| **Bohler’s angle** | Metric, degrees |
| **ASA classification** | 1= ASA I, 2= ASA II, 3= ASA III, 4= ASA IV |
| **Overweight^3^** | Metric, kg/m^2^ |
| **Active smoker** | 0= no, 1= yes |
| **Alcohol abuse** | 0= no, 1= yes |
| **Drug addiction** | 0= no, 1=yes |
| **Arterial hypertonia** | 0=no, 1= yes |
| **Anticoagulation** | 0= no, 1= yes |

^1^time-to-surgery

^2^Minimally invasive approach

^3^BMI >24

## Supplement 2

Model A: Univariable linear regression analyses of independent variables

| **Independent variables** | **Regression coefficient *β*** | ***p*-value** | **95% CI** |
| --- | --- | --- | --- |
| **Age** | -0.007 | 0.929 | -0.166, 0.152 |
| **Sex** | -0.705 | 0.833 | -7.364, 5.954 |
| **TTS^1^** | 1.086 | <0.001 | 0.553, 1.620 |
| **Open Fracture** | 6.484 | 0.150 | -2.405, 15.373 |
| **MIA** | -9.409 | <0.001 | -14.043, -4.774 |
| **Impaired wound healing** | 8.600 | <0.001 | 3.948, 13.253 |
| **Böhler’s angle** | -0.205 | 0.004 | -0.344, -0.067 |
| **ASA classification** | 2.263 | 0.156 | -0.879, 5.405 |
| **Overweight^2^** | 0.556 | 0.073 | -0.054, 1.166 |

^1^time-to-surgery

^2^BMI >24

## Supplement 3

Model B: Univariable binary logistic regression analyses

| **Independent variables** | **Regression coefficient *β*** | ***Wald*** | ***p*-value** | **OR** | **95% CI** |
| --- | --- | --- | --- | --- | --- |
| **Age** | -0.007 | 0.183 | 0.669 | 0.993 | 0.961, 1.026 |
| **Sex** | 0.303 | 0.196 | 0.658 | 1.353 | 0.354, 5.171 |
| **Open Fracture** | 2.374 | 4.261 | 0.039 | 10.737 | 1.127, 102.250 |
| **TTS^1^** | -0.024 | 0.134 | 0.714 | 0.976 | 0.859, 1.110 |
| **MIA^2^** | -0.836 | 1.792 | 0.181 | 0.433 | 0.127, 1.474 |
| **Active smoker** | 0.641 | 0.520 | 0.218 | 1.897 | 0.684, 5.260 |
| **Alcohol abuse** | -1.527 | 1.977 | 0.160 | 0.217 | 0.026, 1.825 |
| **Drug addiction** | 0.140 | 0.034 | 0.853 | 1.150 | 0.261, 5.062 |
| **Arterial hypertonia** | -0.043 | 0.006 | 0.940 | 0.958 | 0.314, 2.919 |
| **Anticoagulation** | 0.134 | 0.022 | 0.883 | 1.143 | 0.194, 6.731 |
| **Overweight^3^** | 1.002 | 3.305 | 0.069 | 2.724 | 0.925, 8.027 |

^1^time-to-surgery

^2^Minimally invasive approach

^3^BMI >24

## Supplement 4

| **Independent variables** | **Validation cohort**  ***n* = 25** |
| --- | --- |
| **Age, mean, median (SD)** | 46.4, 46.0 (13.9) |
| **Male, n** | 22 |
| **Sanders classification, n** |  |
| Type I | 1 |
| Type II | 7 |
| Type III | 8 |
| Type IV | 9 |
| **Open fracture** |  |
| **TTS^1^, mean, median (SD)** | 10.0, 10.0, (3.8) |
| **LOS^2^, mean, median (SD)** | 16.8, 15.0 (5.4) |
| **ORIF^3^, n** | 19 |
| **BMI >24, n** |  |
| **Impaired wound healing** | 7 |
